# Synergistic Anti‐Lung Cancer Effects of Cytarabine and a NCBI‐Registered *Streptococcus* via Modulation of Apoptotic and Survival Pathways

**DOI:** 10.1002/fsn3.72187

**Published:** 2026-08-02

**Authors:** Abbas Asoudeh‐Fard, Farnood Afrakhteh, Zahra Rahimi, Rakhshan Hakimelahi, Mohammad Bagher Nazari, Mohamad Mehdi Nemati, Abbas Fazlinia, Asghar Parsaei

**Affiliations:** ^1^ Institute Galilée University Sorbonne Paris North Paris France; ^2^ Research Center for Pharmaceutical Nanotechnology Tabriz University of Medical Sciences Tabriz Iran; ^3^ Department of Microbiology Islamic Azad University, Rasht Branch Rasht Iran; ^4^ Niko Gene Saba Biotech Company, Rayan Novin Pajohan Company Shiraz Iran; ^5^ Department of Chemistry Shi. C, Islamic Azad University, Shiraz Branch Shiraz Iran; ^6^ Department of Microbiology Islamic Azad University, Shiraz Branch Shiraz Iran; ^7^ Pharmaceutical Sciences Research Center Shiraz University of Medical Sciences Shiraz Iran; ^8^ Department of Chemistry Islamic Azad University, Shiraz Branch Shiraz Iran; ^9^ Department of Biology Zand Institute of Higher Education, Rayan Novin Pajohan Company Shiraz Iran; ^10^ Zand Institute of Higher Education Shiraz Iran

**Keywords:** A549 cells, apoptosis, chemosensitization, cytarabine, lung cancer, probiotic, *Streptococcus thermophilus*

## Abstract

Lung cancer remains one of the leading causes of cancer‐related mortality worldwide, and although Cytarabine (CYT) exhibits anticancer activity, its clinical application is often restricted by dose‐dependent toxicity. In this study, we investigated the synergistic anticancer potential of the NCBI‐registered probiotic *Streptococcus thermophilus* Ab.342 SH (GenBank accession no. PX426457) in combination with CYT against human lung adenocarcinoma A549 cells. Following molecular identification of the probiotic isolate, A549 cells were treated with different concentrations of bacterial cell‐free supernatant (CFS), CYT, or their combination, while human umbilical vein endothelial cells (HUVECs) were used to evaluate selective cytotoxicity. Cell viability was assessed using the MTT assay, apoptosis‐related gene expression was analyzed by qRT‐PCR, and apoptotic cell death was confirmed by Annexin V‐FITC/PI flow cytometry. The CFS alone reduced A549 cell viability in a dose‐dependent manner without significant cytotoxicity toward HUVECs. CYT monotherapy exhibited an IC₅₀ of approximately 40 μg/mL, whereas combination treatment with *S. thermophilus* CFS (OD₆₀₀ = 1.2) reduced the IC₅₀ to 145 ng/mL, representing an approximately 276‐fold dose reduction. Combined treatment significantly upregulated *PTEN, BAX, CASP3, CASP8, CASP9, P53, P21*, and *FAS* expression while suppressing *AKT, mTOR, BCL‐2*, and *IκB*, accompanied by a marked increase in apoptotic cells. These findings demonstrate that *S. thermophilus* Ab.342 SH markedly enhances the anticancer efficacy of CYT while substantially reducing the required drug dosage, supporting the potential application of probiotic‐based adjuvant therapy for safer and more effective lung cancer treatment.

## Graphical abstract

Schematic overview of the synergistic anticancer mechanism between *Streptococcus thermophilus* Ab.342 SH and Cytarabine (CYT) (Ara‐C) in A549 human lung adenocarcinoma cells. The probiotic strain, isolated from traditional dairy samples collected in Fars Province, was cultured to obtain its cell‐free supernatant (CFS) containing bioactive metabolites. A549 and HUVEC cells were exposed to four groups: control, CYT alone, *S. thermophilus* CFS alone, and combination treatment. MTT assay results demonstrated selective cytotoxicity toward A549 cells, with > 96% viability in normal HUVECs, and revealed a dramatic 276‐fold reduction in CYT IC₅₀ (from 40 μg/mL to 145 ng/mL) when combined with the probiotic. qRT‐PCR analysis showed upregulation of pro‐apoptotic genes (Caspase‐3, Caspase‐8, Caspase‐9, Bax, PTEN, P53, P21, and FAS) and downregulation of survival and proliferative markers (*AKT*, *mTOR,* and *Bcl‐2*). Flow cytometry confirmed increased early and late apoptosis with minimal necrosis. The graphical flow summarizes the experimental design and key molecular pathways underlying the observed synergy, illustrating the probiotic's potential as a safe and effective chemosensitizer to enhance CYT efficacy and reduce systemic toxicity in lung cancer therapy.

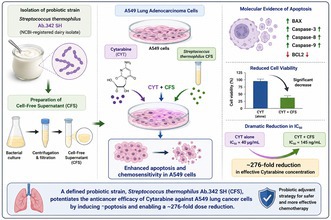

## 1 Introduction

Lung cancer remains the leading cause of cancer‐related deaths globally, accounting for nearly 1.8 million fatalities annually. Among its subtypes, non‐small cell lung cancer (NSCLC) represents approximately 85% of cases and is often diagnosed at advanced stages, where curative treatment options are limited (Hendriks et al. 2024; Smolarz et al. 2025). Current therapeutic approaches include surgery, chemotherapy, targeted therapy, and immunotherapy (Sen et al. 2024; Li et al. 2025). Despite recent advances, the long‐term survival rate of NSCLC patients remains poor, primarily due to drug resistance, tumor heterogeneity, and systemic toxicities associated with chemotherapeutic agents (Tahayneh et al. 2025; Asoudeh‐Fard, Parsaei, et al. 2025). CYT (Ara‐C), a synthetic pyrimidine nucleoside analog, has been extensively used in the treatment of hematological malignancies and exhibits cytotoxic effects on rapidly dividing tumor cells (Jan et al. 2024; Latosińska et al. 2024). CYT functions primarily through incorporation into DNA, thereby inhibiting DNA polymerase activity and triggering apoptosis (Gao et al. 2024; Baghel et al. 2025). Although its potential application in solid tumors, including lung cancer, has been reported, its clinical utility is hampered by limitations such as a short plasma half‐life, dose‐limiting toxicities, and the emergence of resistance mechanisms (Zhao et al. 2024). Therefore, novel strategies that enhance the therapeutic index of CYT while reducing systemic toxicity are urgently needed. In recent years, probiotics—defined as “live microorganisms that, when administered in adequate amounts, confer health benefits to the host”—have emerged as promising adjuncts in oncology (Grujović et al. 2025; Asoudeh‐Fard, Khorrami, et al. 2025). Beyond their well‐established gastrointestinal benefits, specific probiotic strains exhibit anti‐carcinogenic effects through modulation of immune responses, alteration of gut microbiota, reduction of oxidative stress, and regulation of apoptotic and survival signaling pathways (Asoudeh‐Fard, Hosseinzadeh Jahromi, et al. 2025). *Lactic acid bacteria*, particularly *Streptococcus* and *Lactobacillus* species isolated from traditional fermented foods, have demonstrated significant anti‐proliferative and pro‐apoptotic effects against cancer cells. Importantly, these bacteria show minimal or no cytotoxicity toward normal cells, which highlights their potential as safe adjuvants in cancer therapy (Asoudeh‐Fard, Sis, et al. 2025; Mussa Farkhani et al. 2017). Cancer cell fate is tightly regulated by the interplay between apoptotic and survival signaling cascades (Faridvand et al. 2019; Radagdam et al. 2019; Asoudeh‐Fard, Jahromi, et al. 2025). Dysregulation of these pathways contributes not only to tumorigenesis but also to resistance against chemotherapy. Key tumor suppressors such as PTEN (Phosphatase and Tensin Homolog), P53 (Tumor Protein p53), and P21 (Cyclin‐Dependent Kinase Inhibitor 1A) orchestrate cell cycle arrest and DNA damage responses. BAX (BCL2‐Associated X Protein) and Caspases including CASP3 (Caspase‐3), CASP8 (Caspase‐8), and CASP9 (Caspase‐9) are central players in intrinsic and extrinsic apoptotic signaling. Conversely, survival and proliferation are promoted by molecules such as AKT (Protein Kinase B), mTOR (Mechanistic Target of Rapamycin), and IκB (Inhibitor of Nuclear Factor Kappa B), which collectively suppress apoptosis and sustain tumor growth (Asoudeh‐Fard, Najafipour, et al. 2024; Asoudeh‐Fard, Salehi, et al. 2024; Asoudeh‐Fard, Soltanmohammadi, et al. 2025). The *BCL‐2* (*B‐Cell Lymphoma 2*) family further inhibits mitochondrial apoptosis, while death receptor signaling via FAS (*FAS Cell Surface Death Receptor*) provides an extrinsic pathway for apoptosis induction. Targeting this complex molecular network through combined therapeutic modalities represents a rational strategy to enhance anticancer efficacy while minimizing toxicity (Asoudeh‐Fard, Najafipour, et al. 2024; Asoudeh‐Fard, Sis, et al. 2025; Liu et al. 2024; Asoudeh‐Fard, Asoudeh‐Fard, and Parsaei 2025). In this context, we isolated 67 bacterial strains from traditional yogurt samples collected in Fars Province, Iran, and identified a *Streptococcus* strain registered in the NCBI database with potential probiotic properties. This strain was tested for its anti‐cancer activity in combination with CYT against the human lung adenocarcinoma A549 cell line, focusing on synergistic interactions between the two agents. To ensure safety, the probiotic strain was also evaluated on human umbilical vein endothelial cells (HUVECs), confirming its non‐toxic and biocompatible nature. Our research group has previously reported the chemosensitizing effects of another NCBI‐registered *Streptococcus thermophilus* strain (Ab.340 TZ) in combination with doxorubicin against glioblastoma cells, demonstrating modulation of apoptotic and survival pathways (Asoudeh‐Fard, et al. 2026a). Building upon these findings, the present study investigates an entirely different experimental model involving *S. thermophilus* strain Ab.342 SH (GenBank accession no. PX426457), CYT, and human lung adenocarcinoma (A549) cells. This work was designed to determine whether probiotic‐mediated chemosensitization can be extended to a different bacterial isolate, chemotherapeutic agent, and solid tumor model. Importantly, all bacterial isolates, biological samples, experiments, datasets, and molecular analyses reported in this study were generated independently and have not been published previously. **Hypothesis:** We hypothesize that the combination of CYT with a probiotic *Streptococcus* strain will synergistically enhance apoptosis and suppress survival signaling in A549 lung cancer cells, thereby increasing drug sensitivity and allowing for dose reduction. **Objective:** The main objective of this study is to investigate the molecular mechanisms underlying the combined therapeutic effects of CYT and probiotic *Streptococcus*, with specific emphasis on key regulators of apoptosis and survival pathways including *PTEN, AKT, BAX, BCL‐2, CASP3, CASP8, CASP9, P53, P21, FAS, mTOR*, and *IκB*. By integrating a probiotic‐based adjuvant strategy, this research aims to (i) reduce the effective therapeutic dose of CYT, (ii) enhance anticancer efficacy through modulation of apoptotic pathways, and (iii) minimize systemic toxicities, thus offering a safer and more effective therapeutic approach for lung cancer management.

## 2 Materials and Methods

### 2.1 Reagents and Cell Lines

The human lung adenocarcinoma cell line A549 (CCL‐185) and normal HUVECs (ATCC CRL‐1730) were obtained from the National Cell Bank of Iran, Pasteur Institute (Tehran, Iran). Cells were cultured in Dulbecco's Modified Eagle Medium (DMEM; Gibco, UK; Cat. No. 31600‐083) supplemented with 10% fetal bovine serum (FBS; Gibco, UK; Cat. No. 10270106) and 1% Penicillin‐Streptomycin (100 U/mL and 100 μg/mL, respectively; Gibco, UK; Cat. No. 15140122). Cultures were maintained in a humidified incubator at 37°C, 95% relative humidity, and 5% CO_2_ atmosphere. Subculturing was performed at ~80% confluence using 0.25% trypsin‐EDTA (Gibco, UK; Cat. No. 25200056). For functional assays, the following reagents were employed: MTT reagent (Sigma‐Aldrich, UK; Cat. No. M5655), TRIzol reagent (Invitrogen, USA; Cat. No. 15596026) for RNA extraction, iScript cDNA synthesis kit (Bio‐Rad, USA; Cat. No. 1708891), SYBR Green qPCR Master Mix (Ampliqon, Denmark; Cat. No. A322701), and Annexin V‐FITC/PI Apoptosis Detection Kit (eBioscience, Austria; Cat. No. 88‐8005‐74). Flow cytometry analysis was conducted on a BD FACSVerse system (BD Biosciences, USA).

### 2.2 Isolation of *Streptococcus* spp. From Dairy Samples

A total of 67 artisanal dairy products (primarily yogurt) were collected aseptically from 10 rural regions in Fars Province, Iran. For bacterial recovery, 10 g of each sample was homogenized in 90 mL of sterile peptone water, vortexed, and serially diluted. Aliquots were inoculated into de Man, Rogosa, and Sharpe (MRS) broth (Merck, Germany) and incubated at 37°C for 24 h under aerobic conditions. Cultures were subsequently streaked on MRS agar supplemented with 0.05% cysteine and incubated for 48 h. Distinct colonies were picked, sub‐cultured in fresh MRS broth, and preserved for phenotypic and molecular identification. Phenotypic characterization included Gram staining, motility, catalase, and oxidase assays. Colonies that were Gram‐positive, non‐motile, catalase‐ and oxidase‐negative cocci were presumptively identified as Streptococcus spp. For long‐term storage, isolates were suspended in 30% glycerol (w/v) and 10% skim milk (w/v) and stored at −70°C (Asoudeh‐Fard, Beygi, et al. 2024).

### 2.3 Bacterial Isolation and Identification

A total of 67 bacterial strains were isolated from traditional yogurt samples collected from Fars Province, Iran. Samples were cultured on selective media under anaerobic and microaerophilic conditions to facilitate the growth of lactic acid bacteria. Colonies with distinct morphologies were purified by repeated streaking and subjected to Gram staining and catalase testing for preliminary identification. DNA was extracted using a standard phenol‐chloroform method, and the 16S rRNA gene was amplified via polymerase chain reaction (PCR) using universal primers. The PCR products were sequenced, and sequence analysis was performed using BLAST against the NCBI database to confirm bacterial identity. A *Streptococcus* strain exhibiting probiotic potential was selected for further study and registered in NCBI GenBank (Asoudeh‐Fard, Beygi, et al. 2024).

### 2.4 Molecular Identification of *Streptococcus* Isolates

Genomic DNA was extracted using a Qiagen bacterial DNA extraction kit (Qiagen, Germany). Amplification of the 16S rRNA gene was performed using genus‐specific primers (Table 1). PCR was conducted on a Peqlab thermocycler (Erlangen, Germany) with the following profile: initial denaturation at 94°C for 2 min, followed by 32 cycles of denaturation (94°C, 45 s), annealing (55°C, 35 s), and extension (72°C, 1 min), with a final extension at 72°C for 10 min. Amplicons were separated by agarose gel electrophoresis and sequenced commercially (Cinagene, Tehran, Iran). Sequences were compared to NCBI GenBank entries using BLAST analysis. One isolate was confirmed as *S. thermophilus* and deposited in GenBank under the designation *S. thermophilus* strain Ab.342 SH (Accession no. PX426457).

**TABLE 1:** The primer's sequences used in qPCR.

Primer	Amplicon size (bp)	NCBI RefSeq No	Forward (5′to 3′)	Reverse (5′to 3′)
Caspase‐3	145 bp	NM_004346.4	TGGTTCATCCAGTCGCTTTGT	CCCGGGTAAGAATGTGCATAAA
Caspase‐9	132 bp	NM_001229	CTCAGACCAGAGATTCGCAAAC	GCATTTCCCCTCAAACTCTCAA
Caspase‐8	150 bp	NM_001228	GACAGAGCTTCTTCGAGACAC	GCTCGGGCATACAGGCAAAT
AKT	150 bp	NM_005163	CATCACACCACCTGACCAAT	CTCAAATGCACCCGAGAAAT
PTEN	150 bp	NM_000314	TCCCAGTCAGAGGCGCTATG	CAAACTGAGGATTGCAAGTTC
mTOR	150 bp	NM_004958	GACAACAGCCAGGGCCGCAT	ACGCTGCCTTTCTCGACGGC
P21	150 bp	NM_000389	GGTTCATGCCAGCTACTTCC	CCCTTCAAAGTGCCATCTGT
P53	150 bp	NM_000546	TGCGTGTGGAGTATTTGGATG	TGGTACAGTCAGAGCCAACCTC
Fas	150 bp	NM_000043	ATGCTGGGCATCTGGACCC	TCTAGACCAAGCTTTGGATTT
BAX	150 bp	NM_001287.4	ATCCAGGATCGAGCAGGGCG	GGTTCTGATCAGTTCCGGCA
Bcl‐2	150 bp	NM_000657	GTTCCCTTTCCTTCCATCC	GACGGTAGCGACGAGAG
GAPDH	120–110 bp	NM_002046	ATGATGATATCGCCGGCCGCTC	CCCACCATCACGCCCTGG
*Streptococcus* 16S rRNA	500 bp	NC_006449.1	AGAGTTTGATCMTGGCTCAG	ATTACCGCGGCTGCTGG

### 2.5 Cell Culture, Treatment Design, and Cell Viability Assessment

The human lung adenocarcinoma cell line (A549) and normal HUVECs were obtained from the Pasteur Institute of Iran. Both cell lines were cultured in Dulbecco's Modified Eagle Medium (DMEM, Gibco, UK) supplemented with 10% fetal bovine serum (FBS) and 1% penicillin‐streptomycin, and maintained at 37°C in a humidified atmosphere containing 5% CO_2_. Cells were sub‐cultured at 70%–80% confluence, and all experiments were performed using cells in the logarithmic growth phase. The selected *S. thermophilus* strain, previously isolated and identified from traditional yogurt samples of Fars Province, was cultured in MRS broth at 37°C for 24 h. After incubation, cultures were centrifuged at 5000× *g* for 10 min to remove bacterial cells, and the supernatant was collected and filtered through sterile 0.22 μm pore‐size membrane filters (Millipore) to obtain a cell‐free supernatant (CFS) containing secreted metabolites such as short‐chain fatty acids, bacteriocins, and peptides. The sterile CFS was stored at 4°C until used for cell treatment. The selected concentrations of CYT (20–50 μg/mL) were chosen based on previous in vitro studies reporting significant cytotoxic and antiproliferative effects in cancer cells within this concentration range (Zheng et al. 2025), as well as our preliminary dose–response experiments, which confirmed their effectiveness in reducing A549 cell viability while preserving sufficient viable cells for comparative analysis. Similarly, the optical density range of bacterial CFS (OD₆₀₀ = 0.6–1.5) was selected based on previous studies investigating the biological activity of probiotic‐derived CFS in cancer cell models (Asoudeh‐Fard et al. 2026b), together with our preliminary screening experiments. Similarly, the optical density range of bacterial CFS (OD₆₀₀ = 0.6–1.5) was determined based on preliminary screening assays conducted in our laboratory. Among these, OD 1.2 exhibited a moderate but significant inhibitory effect and was therefore selected for combination treatment to evaluate potential synergistic interactions with CYT. For treatment experiments, A549 cells were divided into four experimental groups: (i) untreated control, (ii) CYT (Ara‐C) alone at concentrations of 20, 30, 40, and 50 μg/mL, (iii) *S. thermophilus* CFS alone with optical densities (OD₆₀₀) of 0.6, 0.9, 1.2, and 1.5, and (iv) combination treatment of CYT and bacterial CFS at different concentrations (125 ng/mL + OD 1.2, 135 ng/mL + OD 1.2, 145 ng/mL + OD 1.2, and 155 ng/mL + OD 1.2). To evaluate the biosafety of the probiotic strain, HUVEC cells were treated exclusively with the same concentrations of bacterial CFS. All treatments were conducted for 24 h under standard culture conditions. Cell viability was assessed using the 3‐(4,5‐dimethylthiazol‐2‐yl)‐2,5‐diphenyltetrazolium bromide (MTT) assay. A549 cells were seeded in 96‐well plates at a density of 1 × 10^4^ cells per well and allowed to adhere overnight. After 24 h of treatment with CYT, bacterial CFS, or their combinations, 20 μL of MTT solution (5 mg/mL in PBS) was added to each well and incubated for 4 h at 37°C. The resulting formazan crystals were dissolved in 200 μL of isopropanol, and absorbance was measured at 570 nm using a BioTek ELx808 microplate reader. Cell viability (%) was calculated relative to the untreated control group. Each treatment was performed in triplicate, and the results were expressed as mean ± standard deviation (SD) from three independent experiments to ensure reproducibility and statistical reliability (Ozma et al. 2024).

### 2.6 Quantitative Real‐Time PCR (qRT‐PCR)

For gene expression analysis, A549 cells were seeded in 6‐well plates at a density of 2 × 10^5^ cells per well and allowed to adhere overnight. Cells were then treated with CYT (40 μg/mL), *S. thermophilus* CFS (OD₆₀₀ = 1.2), or the optimized combination treatment (145 ng/mL CYT + OD₆₀₀ = 1.2 CFS) for 24 h under standard culture conditions. After treatment, total RNA was extracted using TRIzol reagent (Invitrogen, USA) according to the manufacturer's protocol. RNA purity and concentration were assessed using a NanoDrop spectrophotometer, ensuring A260/A280 ratios between 1.8 and 2.0, and RNA integrity was verified by agarose gel electrophoresis. Complementary DNA (cDNA) was synthesized from 1 μg of total RNA using the iScript cDNA Synthesis Kit (Bio‐Rad, USA) in a 20 μL reaction volume, following the manufacturer's instructions. Quantitative real‐time PCR (qRT‐PCR) was performed using SYBR Green Master Mix (Ampliqon, Denmark) on a Bio‐Rad iQ5 real‐time PCR system. Specific primers were designed for key apoptotic and survival‐related genes, including *PTEN, AKT, BAX, BCL‐2, CASP3, CASP8, CASP9, P53, P21, FAS, mTOR*, and *IκB*, with *GAPDH* serving as the internal reference gene (see Table 1). Thermal cycling conditions were optimized individually for each gene, consisting of an initial denaturation at 95°C for 3 min, followed by 35 cycles of denaturation at 95°C for 15 s, annealing at 55°C–60°C for 30 s (optimized per primer pair), and extension at 72°C for 30 s. A melting curve analysis was conducted at the end of each run to confirm amplification specificity and the absence of primer‐dimers. All samples were run in technical triplicates, and three independent biological replicates were analyzed. Relative gene expression levels were calculated using the 2^−ΔΔ*Ct*^ method, comparing treated groups to the untreated control. Changes in gene expression were expressed as fold changes, and statistical analyses were performed to evaluate significant differences among treatments (Asoudeh‐Fard and Pavon‐Djavid 2025).

### 2.7 Flow Cytometry for Apoptosis Detection

Apoptosis was assessed using the Annexin V‐FITC/PI kit. After 24 h of treatment with CYT, CFS, or the combination, cells were harvested, washed twice with PBS, and resuspended in binding buffer. Cells were stained with Annexin V‐FITC and PI for 15 min in the dark. Analysis was performed on a BD FACSVerse cytometer with FlowJo software. Populations were classified as viable (Annexin V^−^/PI^−^), early apoptotic (Annexin V^+^/PI^−^), late apoptotic (Annexin V^+^/PI^+^), or necrotic (Annexin V^−^/PI^+^) (Asoudeh‐Fard, Mohkam, et al. 2025).

### 2.8 Statistical Analysis

All experiments were performed in triplicate biological replicates, each with technical triplicates. Data were expressed as mean ± SD. Normality was checked by the Shapiro–Wilk test. Group comparisons were made by one‐way ANOVA followed by Tukey's post hoc test, while unpaired two‐tailed Student's *t*‐test was used for two‐group comparisons. Statistical significance was set at *p* < 0.05. Analyses were conducted using SPSS v24 (IBM, USA).

## 3 Results

### 3.1 Growth‐Inhibitory Effects of *S. thermophilus* Strain ab.342 SH and Cytarabine

**FIGURE 1:**
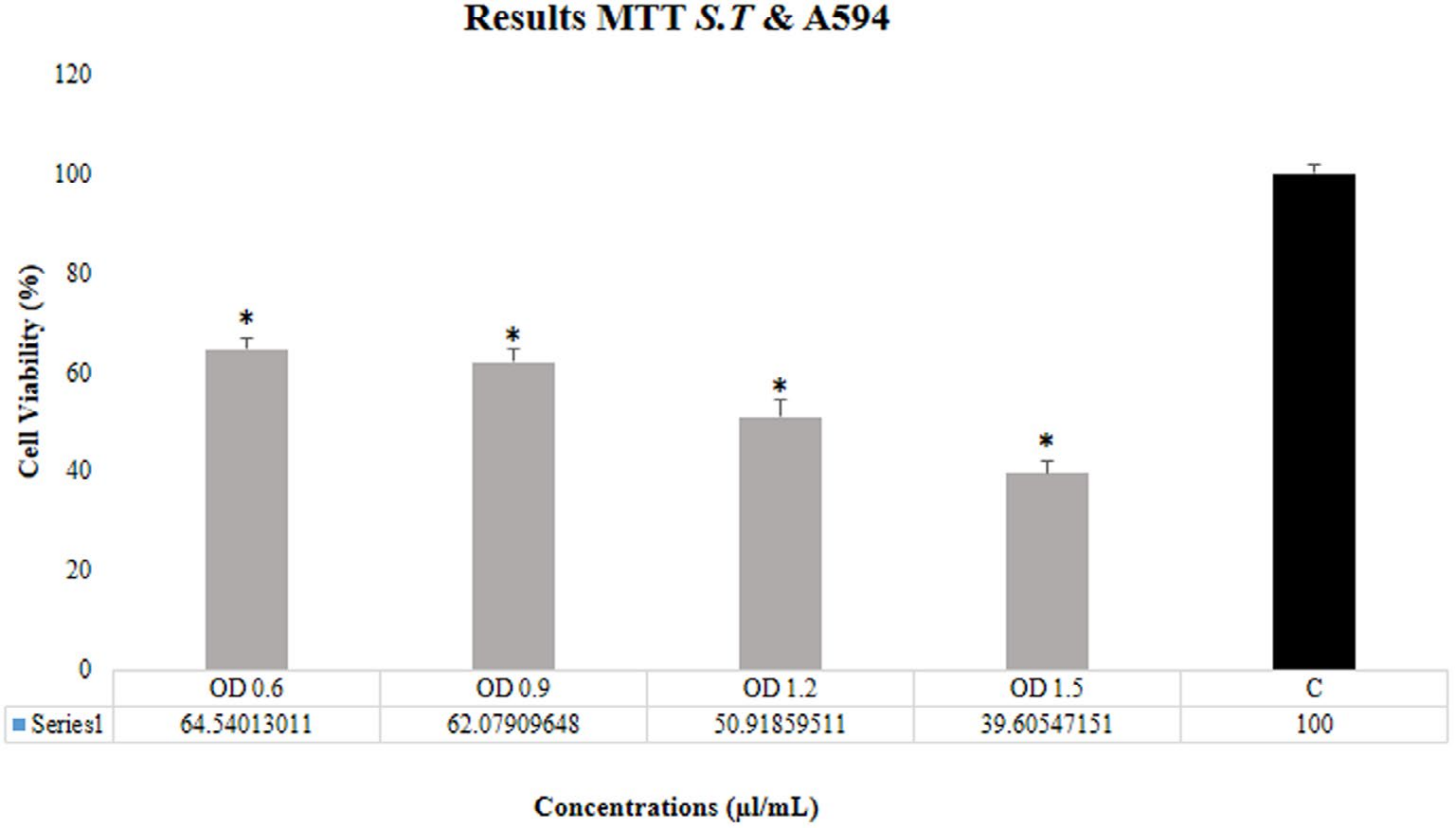
Effect of *Streptococcus thermophilus* Ab.342 SH CFS on the viability of A594 cervical cancer cells. Cells were treated with increasing bacterial concentrations (OD_600_: 0.6, 0.9, 1.2, and 1.5; 10 μL/mL) for 24 h, and cell viability was assessed using the MTT assay. Data are presented as mean ± SD from three independent experiments. Statistical significance versus untreated controls is indicated as *p < 0.05, **p < 0.01, ***p < 0.001.

**FIGURE 2:**
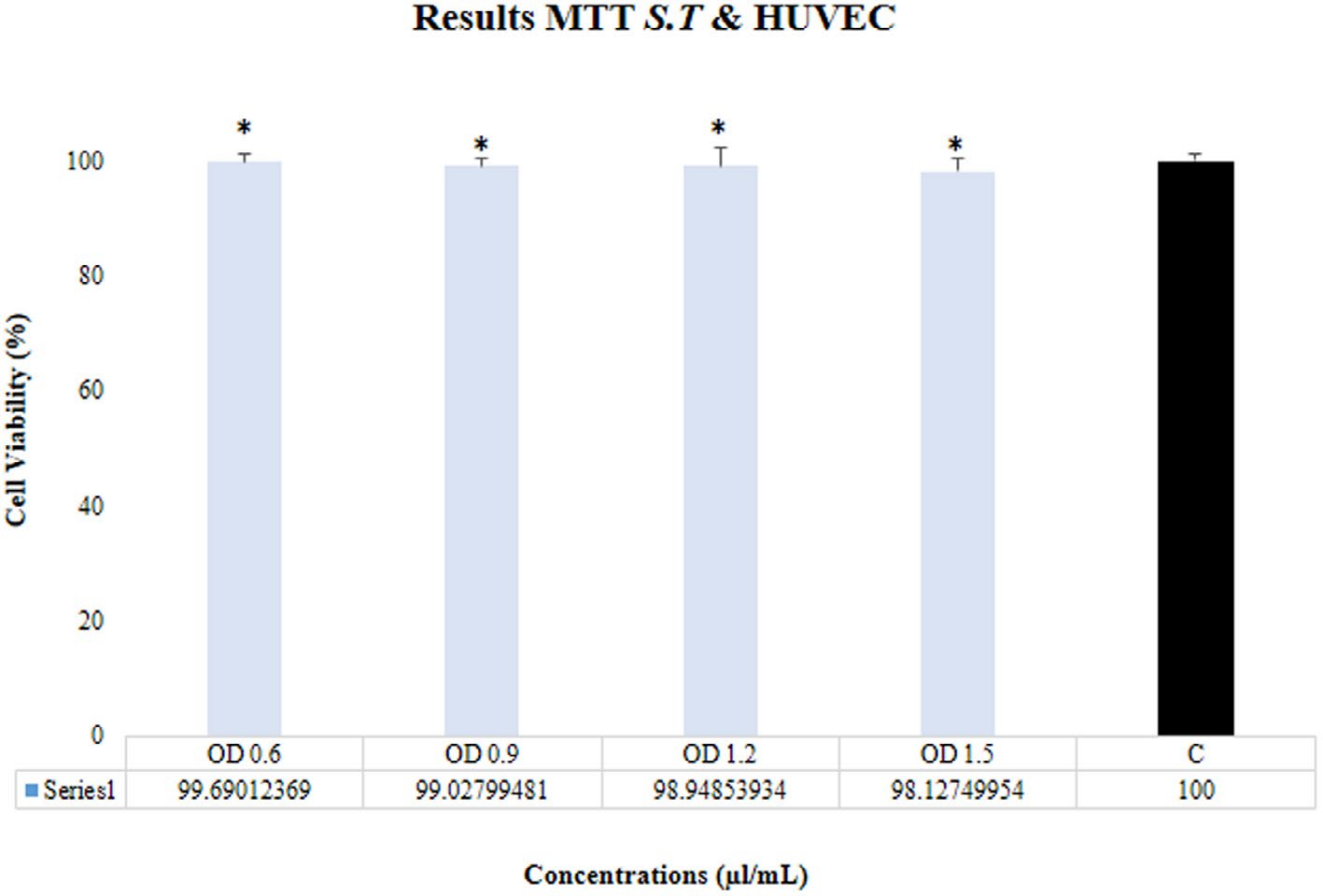
Effect of *Streptococcus thermophilus* Ab.342 SH CFS on the viability of human umbilical vein endothelial cells (HUVECs). Cells were incubated with different bacterial concentrations (OD_600_: 0.6, 0.9, 1.2, and 1.5; 10 μL/mL) for 24 h, and cell survival was determined by MTT assay. Data represent mean ± SD of three independent experiments. Statistical differences compared with untreated controls are shown as *p < 0.05, **p < 0.01, ***p < 0.001.

**FIGURE 3:**
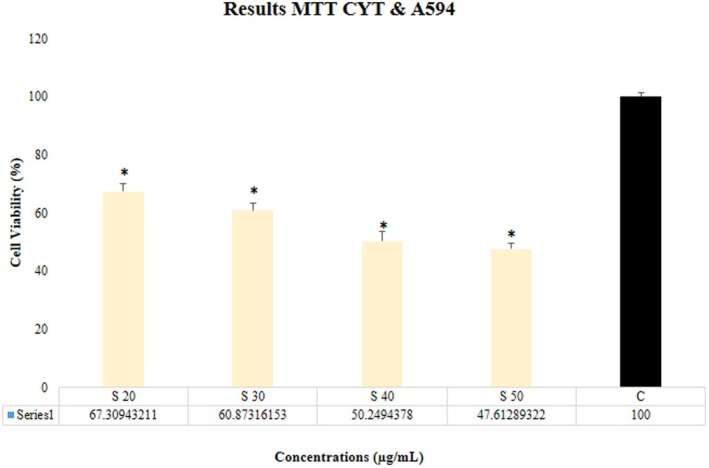
Cytotoxic effects of Cytarabine (CYT) on A594 cells. Cells were treated with increasing concentrations of CYT (20, 30, 40, 50 μg/mL) for 24 h, and cell viability was evaluated using the MTT assay. Results are expressed as mean ± SD from three independent biological replicates. Statistical significance versus untreated controls is indicated as *p < 0.05, **p < 0.01, ***p < 0.001.

**FIGURE 4:**
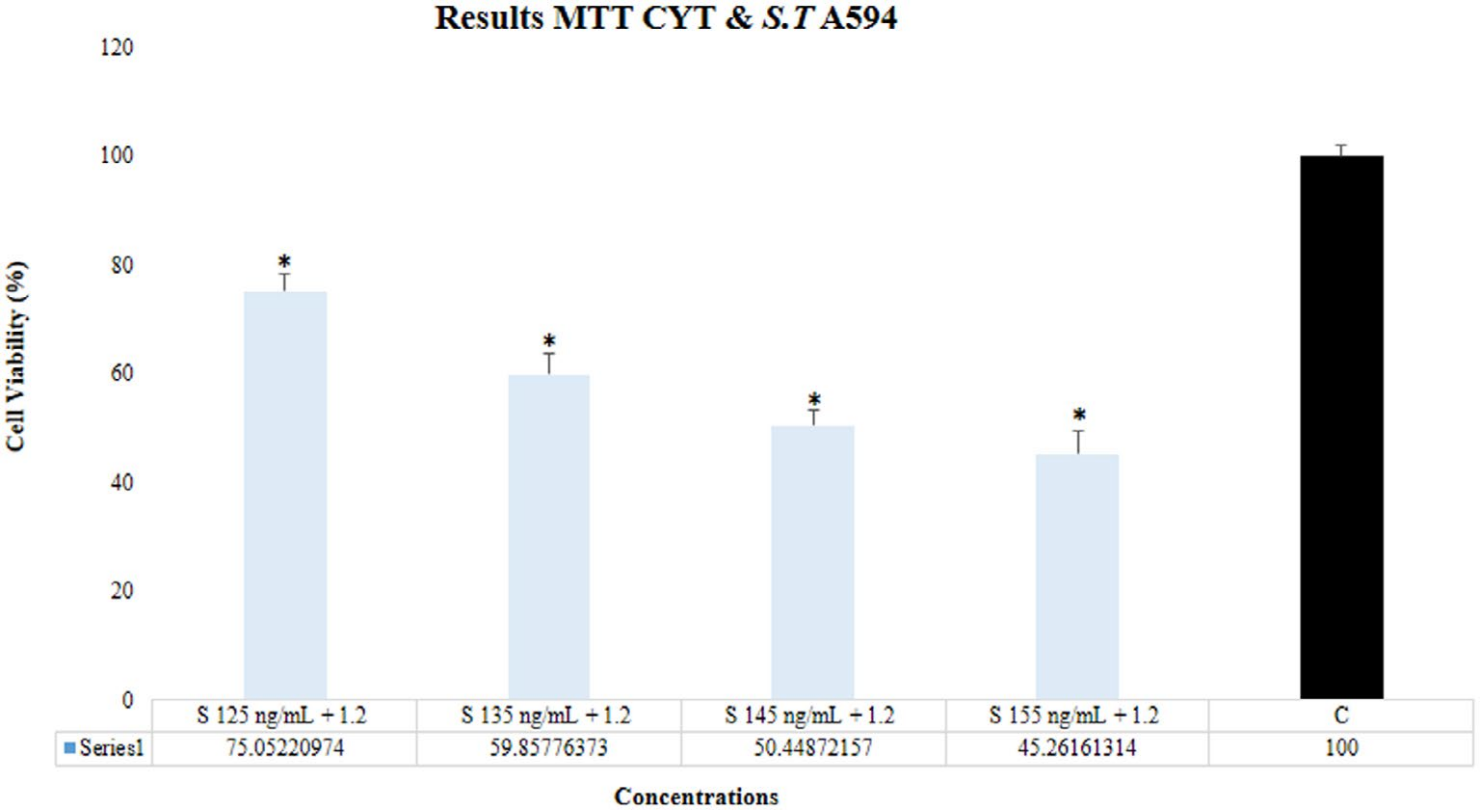
Combined cytotoxic effects of *Streptococcus thermophilus* Ab.342 SH CFS and Cytarabine (CYT) on A594 cells. Cells were co‐treated with *S. thermophilus* CFS (OD_600_ = 1.2) and various CYT concentrations (125, 135, 145, 155 ng/mL) for 24 h. Cell viability was measured using the MTT assay. Data represent mean ± SD from three independent experiments. Significant differences compared with untreated controls are shown as *p < 0.05, **p < 0.01, ***p < 0.001.

The cytotoxic potential of *S. thermophilus* strain Ab.342 SH was initially evaluated in A549 lung adenocarcinoma cells and compared with normal HUVEC endothelial cells using the MTT assay. Treatment with increasing concentrations of bacterial CFS (OD₆₀₀ = 0.6, 0.9, 1.2, and 1.5; 10 μL/mL) produced a dose‐dependent decrease in A549 cell viability, ranging from 64.54% ± 2.12% at OD₆₀₀ = 0.6 to 39.60% ± 2.15% at OD₆₀₀ = 1.5. The half‐maximal inhibitory concentration (IC₅₀) was determined to be OD₆₀₀ = 1.2, corresponding to 50.91% ± 2.31% cell survival (Figure 1). In contrast, HUVECs maintained high viability (> 96%) across all tested doses (range: 99.69% ± 0.14% to 98.12% ± 0.30%), demonstrating selective cytotoxicity of *S. thermophilus* toward malignant cells while sparing normal endothelial cells (Figure 2). Baseline chemosensitivity of A549 cells was established by treatment with increasing concentrations of CYT (20–50 μg/mL), resulting in a progressive reduction in viability from 67.30% ± 1.26% at 20 μg/mL to 47.61% ± 1.62% at 50 μg/mL, with an IC₅₀ of approximately 40 μg/mL (50.24% ± 2.10%) (Figure 3). Remarkably, co‐administration of *S. thermophilus* CFS at OD₆₀₀ = 1.2 with CYT dramatically enhanced cytotoxic efficacy, reducing the IC₅₀ of CYT to 145 ng/mL (50.44% ± 2.10%), representing over a 276‐fold dose reduction while maintaining comparable anti‐proliferative effects (Figure 4). These results indicate a dose‐sparing and chemosensitizing effect of *S. thermophilus* , supporting its potential as an adjuvant to reduce systemic toxicity associated with high‐dose chemotherapy.

### 3.2 Differential Gene Expression in Response to Treatments

**FIGURE 5:**
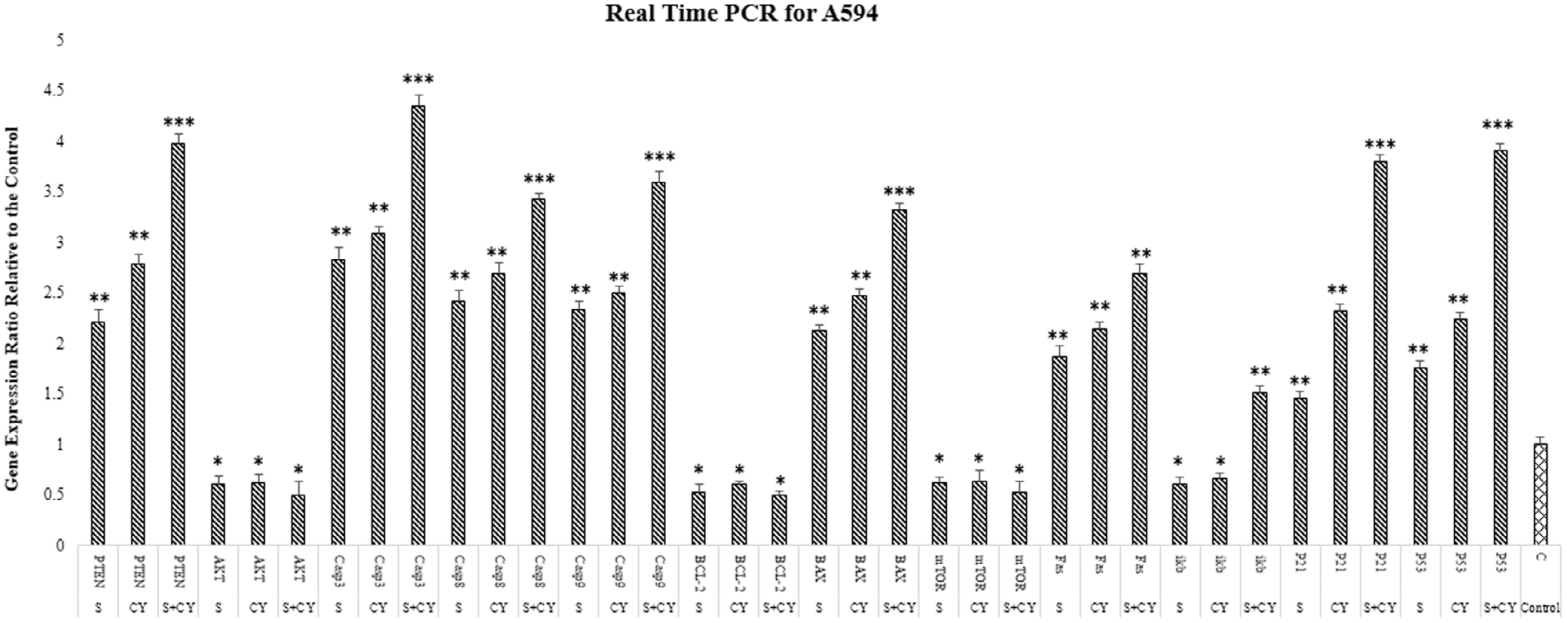
Expression of apoptosis‐related genes in A594 cells after treatment with *Streptococcus thermophilus* CFS and CYT. Cells were treated for 24 h with CYT (40 μg/mL), *S. thermophilus* CFS (OD_600_ = 1.2), or their combination at IC_50_ concentrations. qRT‐PCR analysis revealed upregulation of pro‐apoptotic genes (*Caspase‐3, Caspase‐8, Caspase‐9, Bax, PTEN, P53, P21, IκB, Fas*) and downregulation of anti‐apoptotic/survival genes (*Bcl‐2, AKT, mTOR*). Data represent mean ± SD from three independent experiments. Statistical significance versus controls: *p < 0.05, **p < 0.01, ***p < 0.001. *S*, *S. thermophilus* ; *CY*, Cytarabine; *CY* + *S*, combination.

**FIGURE 6:**
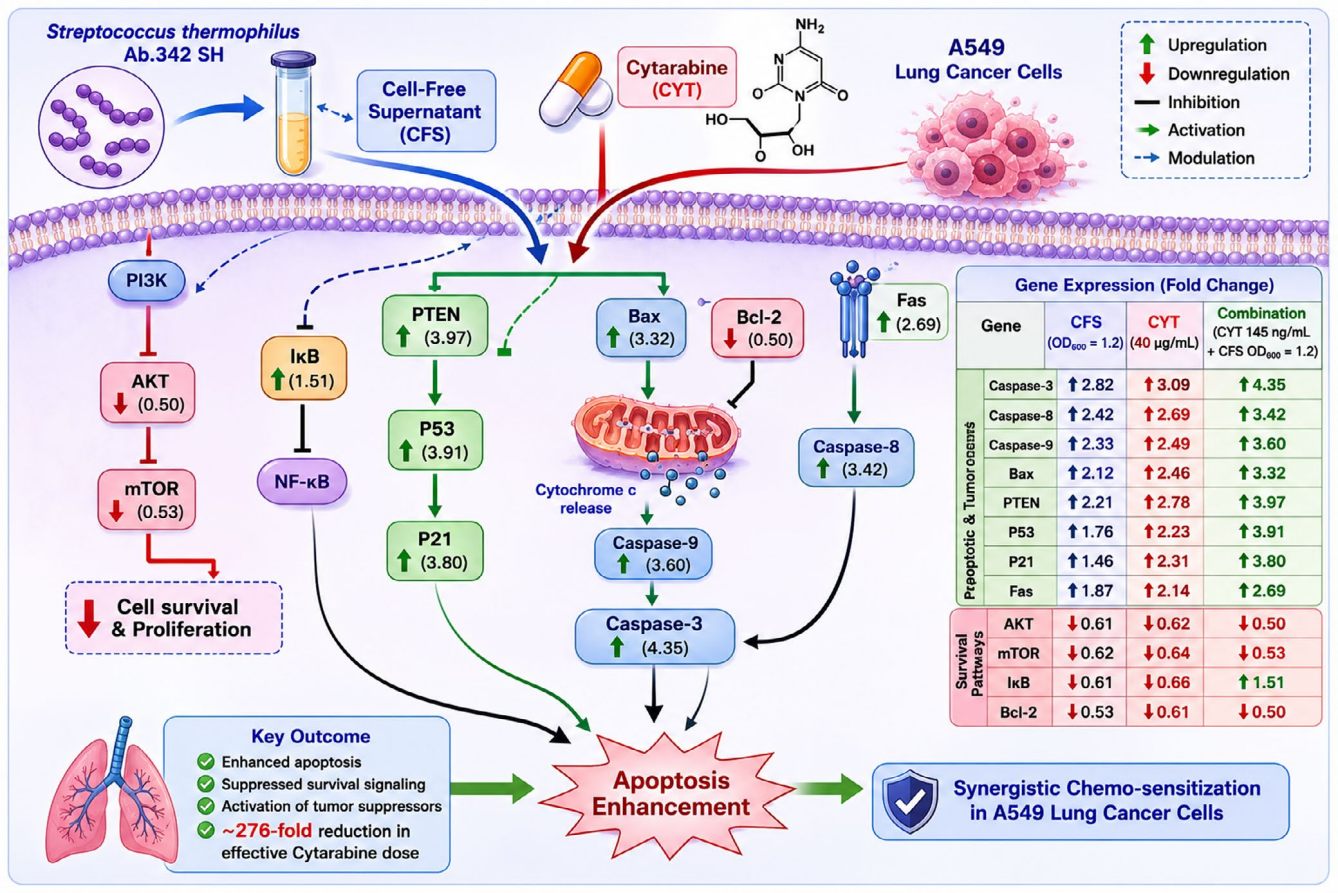
Proposed molecular mechanism underlying the synergistic anticancer activity of *Streptococcus thermophilus* Ab.342 SH cell‐free supernatant (CFS) in combination with Cytarabine (CYT) in A549 lung adenocarcinoma cells. Treatment with the combined regimen markedly enhanced apoptotic signaling through activation of both the intrinsic and extrinsic apoptosis pathways. Compared with either treatment alone, the combination resulted in increased expression of *PTEN* (↑3.97‐fold), *P53* (↑3.91‐fold), *P21* (↑3.80‐fold), *Bax* (↑3.32‐fold), *Fas* (↑2.69‐fold), *Caspase‐8* (↑3.42‐fold), *Caspase‐9* (↑3.60‐fold), and *Caspase‐3* (↑4.35‐fold), while suppressing the survival‐related genes *AKT* (↓0.50‐fold), *mTOR* (↓0.53‐fold), and *Bcl‐2* (↓0.50‐fold). *IκB* showed increased expression (↑1.51‐fold), suggesting modulation of the *NF‐κB* signaling pathway. Collectively, these molecular alterations promote apoptosis, inhibit cell survival and proliferation, and provide a mechanistic basis for the enhanced chemosensitivity and substantial reduction in the effective Cytarabine concentration observed following combination treatment.

To elucidate the molecular mechanisms underlying the observed cytotoxicity, qRT‐PCR analysis was performed on A549 cells treated for 24 h with IC₅₀ concentrations of *S. thermophilus* CFS (OD₆₀₀ = 1.2), CYT (40 μg/mL), or the optimized combination (CYT 145 ng/mL + OD₆₀₀ = 1.2 CFS). Treatment with *S. thermophilus* alone significantly increased the expression of selected pro‐apoptotic and tumor suppressor genes, including *Caspase‐3* (2.82‐fold), *Caspase‐8* (2.42‐fold), *Caspase‐9* (2.33‐fold), *Bax* (2.12‐fold), *PTEN* (2.21‐fold), *P53* (1.76‐fold), and *P21* (1.46‐fold). Concurrently, survival‐associated genes were downregulated: *AKT* (0.61‐fold), *mTOR* (0.62‐fold), *IκB* (0.61‐fold), and *Bcl‐2* (0.53‐fold), whereas Fas expression was moderately increased (1.87‐fold). CYT monotherapy elicited a more pronounced induction of pro‐apoptotic genes, with *Caspase‐3* (3.09‐fold), *Caspase‐8* (2.69‐fold), *Caspase‐9* (2.49‐fold), *Bax* (2.46‐fold), *PTEN* (2.78‐fold), *P53* (2.23‐fold), and *P21* (2.31‐fold), alongside downregulation of *AKT* (0.62‐fold), *mTOR* (0.64‐fold), *IκB* (0.66‐fold), and *Bcl‐2* (0.61‐fold), while *Fas* increased to 2.14‐fold. The combination treatment produced the most pronounced changes in expression of the selected apoptotic and survival genes, with strong induction of *Caspase‐3* (4.35‐fold), *Caspase‐8* (3.42‐fold), *Caspase‐9* (3.60‐fold), *Bax* (3.32‐fold), *PTEN* (3.97‐fold), *P53* (3.91‐fold), and *P21* (3.80‐fold). Survival pathways were simultaneously suppressed to their lowest levels: AKT (0.50‐fold), *mTOR* (0.53‐fold), and *Bcl‐2* (0.50‐fold). Notably, *Fas* expression peaked under combination therapy (2.69‐fold), while *IκB* increased to 1.51‐fold, suggesting a potential compensatory mechanism within *NF‐κB* signaling (Figures 5, 6). These data indicate that both agents individually modulate apoptotic and pro‐survival gene expression, whereas the combination synergistically enhances both intrinsic (*Bax/Caspase‐9*) and extrinsic (*Fas/Caspase‐8*) apoptosis, reinforces tumor suppressor activation (*PTEN, P53, P21*), and more effectively inhibits proliferative signaling (*AKT/mTOR/Bcl‐2*), providing a coherent mechanistic explanation for the observed dose‐sparing cytotoxicity. Statistical analysis by one‐way ANOVA confirmed significance (*p* < 0.05, *p* < 0.01, *p* < 0.001).

### 3.3 Apoptotic Profiling by Flow Cytometry

**FIGURE 7:**
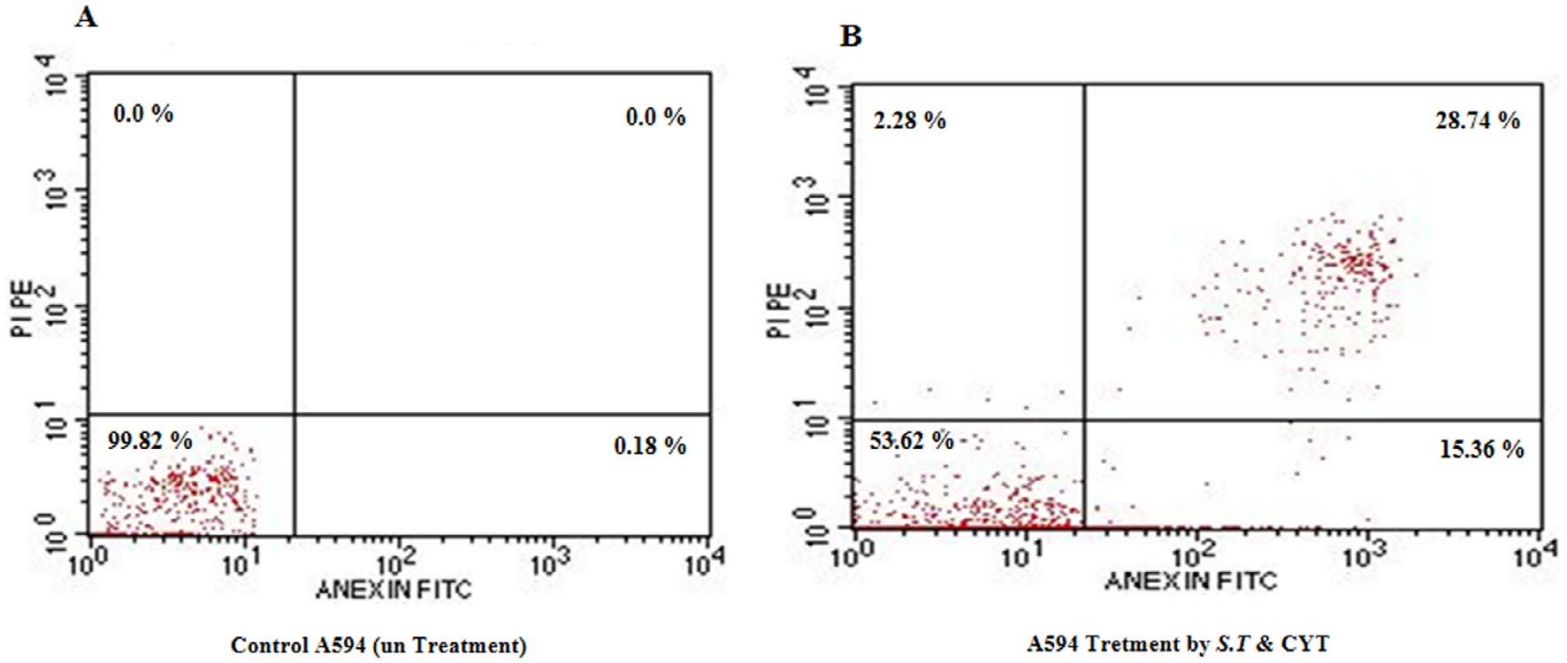
Flow cytometry analysis of apoptosis in A594 cells after co‐treatment with *Streptococcus thermophilus* CFS and CYT. Cells were incubated for 24 h with CYT (145 ng/mL) and *S. thermophilus* (OD_600_ = 1.2), then stained with Annexin V‐FITC/PI. Dot plots indicate viable cells (Annexin^−^/PI^−^), early apoptotic (Annexin^+^/PI^−^), late apoptotic (Annexin^+^/PI^+^), and necrotic cells (Annexin^−^/PI^+^). Representative results from three independent experiments show a marked increase in apoptosis relative to controls.

**FIGURE 8:**
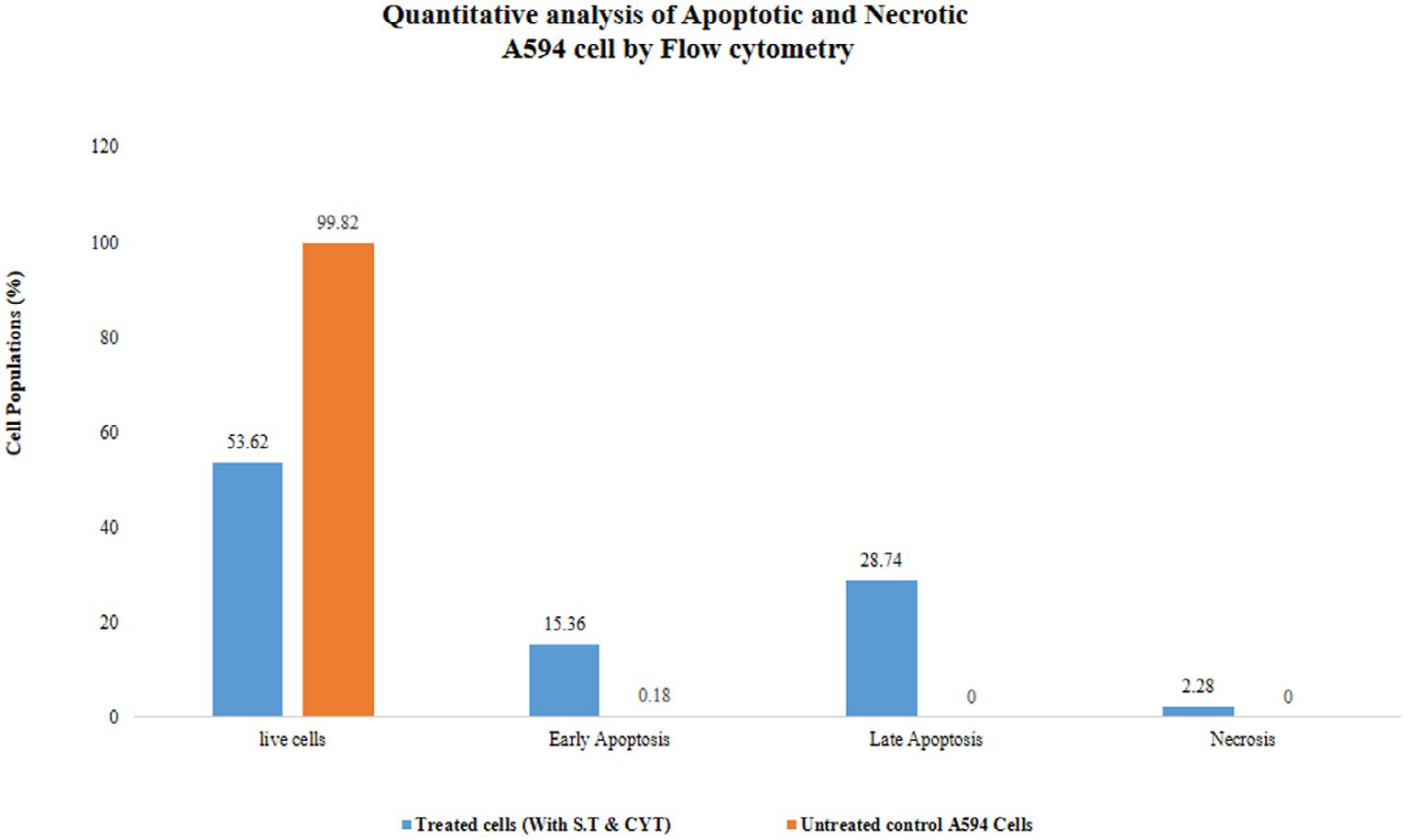
Quantification of apoptotic and necrotic populations in A594 cells after combined treatment with *Streptococcus thermophilus* CFS and CYT. Cells were treated for 24 h with CYT (145 ng/mL) and *S. thermophilus* (OD_600_ = 1.2) at IC_50_. Flow cytometry revealed increases in early apoptosis (15.36%) and late apoptosis (28.74%) compared with controls (2.28%), while necrosis remained minimal. Data are presented as mean ± SEM from three independent replicates. Statistical significance: *p < 0.05, confirming apoptosis as the predominant cytotoxic mechanism.

To validate apoptosis as the primary mode of cell death, Annexin V‐FITC/PI double staining was performed followed by flow cytometric analysis. A549 cells treated with the combination of CYT (145 ng/mL) and *S. thermophilus* (OD_600_ = 1.2) exhibited a substantial increase in apoptotic populations relative to untreated controls. Early apoptotic cells (Annexin V^+^/PI^−^) accounted for 15.36%, late apoptotic cells (Annexin V^+^/PI^+^) for 28.74%, whereas necrotic cells (Annexin V^−^/PI^+^) remained minimal at 2.28%. Analysis across three independent experiments (*n* = 3) confirmed a statistically significant elevation in apoptosis within the combination group (*p* < 0.05) (Figures 7, 8). These findings corroborate transcriptional data, indicating that the CYT– *S. thermophilus* complex preferentially induces apoptotic rather than necrotic cell death in A549 cells.

## 4 Discussion

The present study demonstrates, for the first time, that the NCBI‐registered probiotic strain *S. thermophilus* Ab.342 SH can synergize with CYT to exert a potent anticancer effect against A549 human lung adenocarcinoma cells. The NCBI‐registered probiotic strain S. thermophilus Ab.342 SH can enhance the anticancer efficacy of CYT and act as a potential chemosensitizing adjuvant against A549 human lung adenocarcinoma cells. This approach is novel in multiple aspects, including the use of a molecularly validated, well‐characterized probiotic strain, the evaluation of selective cytotoxicity toward malignant cells while sparing normal HUVECs, and the unprecedented demonstration of a dramatic dose‐sparing effect, reducing the IC₅₀ of CYT from 40 μg/mL to 145 ng/mL (~276‐fold, Figures 1, 2, 3). Specifically, the IC₅₀ of CYT alone in A549 cells was approximately 40 μg/mL, whereas in the presence of *S. thermophilus* CFS (OD₆₀₀ = 1.2), a comparable level of growth inhibition was achieved at a substantially lower CYT concentration of 145 ng/mL. This corresponds to an approximately 276‐fold reduction in the effective CYT concentration required to produce a similar level of growth inhibition, suggesting a marked chemosensitizing effect of the probiotic‐derived metabolites rather than an alteration of the intrinsic pharmacological potency of CYT. Similar dose‐sparing effects resulting from combination therapies have been reported for other probiotic–chemotherapeutic combinations, although the magnitude of reduction observed in the present study appears to be substantially greater (Asoudeh‐Fard, Salehi, et al. 2024; Asoudeh‐Fard, Asoudeh‐Fard, and Parsaei 2025; Vinothkanna et al. 2024). Nevertheless, the observed reduction should be interpreted within the context of an in vitro experimental model, and additional pharmacological synergy analyses together with in vivo validation are required to confirm its translational significance. Our results revealed that *S. thermophilus* alone moderately inhibits A549 cell viability while maintaining high HUVEC survival (> 96%, Figures 1, 2), confirming the selective anticancer potential of this strain. Importantly, treatment of normal HUVECs with the same concentrations of *S. thermophilus* CFS resulted in consistently high cell viability (> 96%), indicating minimal cytotoxicity toward normal endothelial cells. These findings confirm the selective anticancer potential of the probiotic, which preferentially targets malignant A549 cells while sparing healthy cells, supporting both the safety and translational relevance of this chemo‐sensitizing approach. This finding aligns with previous reports where lactic acid bacteria, including *S. thermophilus* , exerted anticancer effects primarily in colorectal, gastric, and breast cancer models via secreted metabolites such as bacteriocins, short‐chain fatty acids (SCFAs), and bioactive peptides (Vinothkanna et al. 2024; D'Amore et al. 2025; Ghosh et al. 2025; Asoudeh‐Fard, Yeylagh‐Beygi, et al. 2025). Unlike prior studies that largely focused on general probiotic mixtures or non‐characterized strains, our work demonstrates that a fully defined, NCBI‐registered strain can act as a chemo‐sensitizer, substantially enhancing the efficacy of CYT while minimizing exposure to toxic doses. At the molecular level, transcriptomic profiling revealed that the combination of CYT and *S. thermophilus* strongly upregulated pro‐apoptotic genes, including *Caspase‐3, Caspase‐8*, *Caspase‐9, Bax, PTEN, P53*, and *P21*, while simultaneously suppressing survival‐associated genes such as *AKT, mTOR*, and *Bcl‐2* (Figures 4, 5). Notably, Fas expression was elevated, indicating activation of the extrinsic apoptotic pathway in conjunction with intrinsic *Bax/Caspase‐9*‐mediated apoptosis, while a moderate increase in *IκB* suggested nuanced regulation of *NF‐κB* signaling. These findings are corroborated by flow cytometric analysis, which confirmed significant increases in early and late apoptotic populations with minimal necrosis (Figure 6). Previous research has shown that probiotics can modulate apoptosis‐related pathways in cancer cells (Asoudeh‐Fard et al. 2026a; Raveendran et al. 2026; Tavallaei et al. 2025), but the comprehensive and synergistic activation of both intrinsic and extrinsic apoptosis observed here, in combination with tumor suppressor reinforcement and proliferative pathway inhibition, represents a novel mechanism that has not been previously demonstrated in lung adenocarcinoma models. The translational and clinical advantages of this approach are substantial. First, the selective cytotoxicity toward cancer cells with negligible effects on normal endothelial cells underscores both the safety and biocompatibility of the probiotic adjuvant. Second, the dramatic reduction in CYT dosage (~276‐fold) may mitigate dose‐limiting toxicities, including myelosuppression and gastrointestinal complications, thereby improving patient tolerability. Third, the coordinated modulation of multiple apoptotic and survival pathways provides a mechanistic rationale for the observed synergy and establishes a robust framework for preclinical validation. Provides a mechanistic rationale for the observed chemo‐sensitizing and enhanced anticancer effects. Fourth, using a molecularly characterized, NCBI‐registered strain enhances reproducibility and translational potential, facilitating future clinical development. Lastly, the integration of functional, cytotoxic, and transcriptomic analyses provides a comprehensive mechanistic understanding that strengthens the evidence base for probiotic‐mediated chemo‐sensitization. Compared with prior literature, our findings represent a significant advance by demonstrating that a defined probiotic strain can directly enhance chemotherapeutic efficacy in lung cancer, rather than relying on uncharacterized or mixed strains. Although our research group previously reported the chemosensitizing effect of another NCBI‐registered *S. thermophilus* strain (Ab.340 TZ) in combination with doxorubicin against glioblastoma cells (Asoudeh‐Fard et al. 2026a), the present study represents an independent investigation. Specifically, this work employed a different probiotic isolate ( *S. thermophilus* Ab.342 SH; GenBank accession no. PX426457), a different chemotherapeutic agent (CYT), and a distinct cancer model (A549 lung adenocarcinoma cells). Furthermore, all bacterial isolates, biological materials, cell culture experiments, MTT assays, qRT‐PCR analyses, flow cytometry experiments, datasets, and statistical analyses presented in this manuscript were generated independently and have not been published previously. These findings therefore extend our previous observations by demonstrating that probiotic‐mediated chemosensitization is not restricted to a single probiotic strain, chemotherapeutic drug, or tumor type, but may represent a broader therapeutic strategy for enhancing anticancer efficacy. Moreover, the quantitative assessment of both apoptotic and survival pathways allows a deeper understanding of the molecular interplay underlying synergistic cytotoxicity, which has been largely unexplored in earlier studies. This work therefore positions probiotics not merely as supportive agents but as active modulators capable of expanding the therapeutic window of conventional chemotherapeutics. In conclusion, this study highlights the novelty, mechanistic insight, and translational relevance of employing *S. thermophilus* Ab.342 SH as a chemo‐sensitizing adjuvant in lung adenocarcinoma therapy, providing a compelling rationale for future in vivo validation and clinical translation. Further pharmacological modeling is required to confirm formal synergy. In addition, it is important to emphasize that these findings are currently limited to in vitro conditions, and in vivo validation using appropriate animal models, such as lung cancer xenografts, is essential to confirm the observed dose‐reduction effect, therapeutic efficacy, and safety profile under physiologically relevant conditions. Such studies will be critical to establish the true translational potential of this chemo‐sensitizing strategy. The integration of selective cytotoxicity, profound dose reduction, and coordinated regulation of apoptotic and survival pathways underscores the potential of defined probiotic–chemotherapy combinations to improve patient outcomes and reduce systemic toxicity in precision oncology.

## 5 Conclusion

In conclusion, the combination of *S. thermophilus* Ab.342 SH with CYT exhibits potent chemosensitizing and enhanced anticancer activity against A549 lung adenocarcinoma cells under in vitro conditions. The combination dramatically enhances apoptosis by modulating both intrinsic and extrinsic pathways, upregulating tumor suppressors (*PTEN, P53, P21*), and suppressing survival signals (*AKT/mTOR/Bcl‐2*). Critically, this strategy allows a ~276‐fold reduction in CYT dosage, highlighting a clear clinical advantage in minimizing chemotherapy‐associated toxicity. These findings support the use of *S. thermophilus* as a safe and effective chemosensitizing adjuvant with potential translational relevance in lung cancer treatment. However, further validation in animal models is necessary to confirm these findings and establish clinical applicability.

## 6 Limitations and Future Directions

Despite these promising results, several limitations must be acknowledged. First, the study was conducted in vitro using a single NSCLC cell line (A549) and normal HUVECs, which may limit the generalizability of the findings. A549 cells were selected as a well‐established and widely used model of human lung adenocarcinoma for initial mechanistic and therapeutic evaluation. However, validation in additional NSCLC cell lines with distinct genetic backgrounds (such as EGFR‐mutant or KRAS‐mutant models), as well as patient‐derived primary tumor cells, is necessary to confirm the broader applicability and translational relevance of the observed chemosensitizing effects. Furthermore, although the combination treatment demonstrated a substantial reduction in the effective CYT dose and enhanced apoptotic responses, formal pharmacological synergy was not quantified using Combination Index (CI) analysis or isobologram methods such as the Chou–Talalay approach. Future studies incorporating these quantitative models are necessary to precisely characterize the nature and strength of the interaction. Second, in vivo studies, particularly using tumor xenograft models, are essential to validate the observed chemo‐sensitizing effect, confirm the significant dosereduction under physiological conditions, and evaluate pharmacokinetics, biodistribution, therapeutic efficacy, and systemic safety of the combination therapy. Third, while the study identifies strong transcriptional changes in apoptotic and survival genes, the precise secreted factors mediating chemosensitization—such as β‐galactosidase, bacteriocins, or short‐chain fatty acids—require further characterization. Future studies could also explore optimal dosing regimens, delivery systems for the probiotic, and combination with other chemotherapeutic agents to broaden therapeutic applicability. Finally, long‐term effects and immunomodulatory impacts of *S. thermophilus* administration in cancer patients remain to be evaluated.

## Author Contributions

**Rakhshan Hakimelahi:** writing – review and editing. **Mohammad Bagher Nazari:** writing – review and editing. **Zahra Rahimi:** methodology. **Asghar Parsaei:** writing – original draft. **Abbas Asoudeh‐Fard:** conceptualization, writing – original draft. **Farnood Afrakhteh:** methodology. **Abbas Fazlinia:** writing – review and editing. **Mohamad Mehdi Nemati:** investigation.

## Funding

The authors have nothing to report.

## Ethics Statement

The study does not contain any experiments on humans or animals.

## Conflicts of Interest

The authors declare no conflicts of interest.

## Data Availability

The 16S rRNA gene sequence of *Streptococcus thermophilus* strain Ab.342 SH has been deposited in the NCBI GenBank database under accession number PX426457 and is publicly available. The remaining datasets generated and/or analyzed during the current study are available from the corresponding author upon reasonable request.
